# New Data on Native and Alien Vascular Flora of Sicily (Italy): New Findings and Updates

**DOI:** 10.3390/plants12091743

**Published:** 2023-04-23

**Authors:** Salvatore Cambria, Dario Azzaro, Orazio Caldarella, Michele Aleo, Giuseppe Bazan, Riccardo Guarino, Giancarlo Torre, Antonia Egidia Cristaudo, Vincenzo Ilardi, Alfonso La Rosa, Valentina Lucia Astrid Laface, Fabio Luchino, Francesco Mascia, Pietro Minissale, Saverio Sciandrello, Luca Tosetto, Gianmarco Tavilla

**Affiliations:** 1 Department of Biological, Geological and Environmental Sciences, University of Catania, Via A. Longo 19, 95125 Catania, Italy; azzaro.dario@gmail.com (D.A.); acristau@unict.it (A.E.C.); s.sciandrello@unict.it (S.S.); gianmarco.tavilla@phd.unict.it (G.T.); 2 Independent Researcher, Via Maria SS. Mediatrice 38, 90129 Palermo, Italy; orazio.caldarella@gmail.com; 3 Independent Researcher, Via S. Safina, 91100 Trapani, Italy; michele.aleo@libero.it; 4 Department of Biological, Chemical and Pharmaceutical Sciences and Technologies (STEBICEF), University of Palermo, 90123 Palermo, Italy; giuseppe.bazan@unipa.it (G.B.); riccardo.guarino@unipa.it (R.G.); vincenzo.ilardi@unipa.it (V.I.); 5 Stiftung Pro Artenvielfalt^®^, Meisenstraße 65, 33607 Bielefeld, Germany; giancarlotorre@hotmail.it; 6 Cooperativa Silene, Via V. D’Ondes Reggio 8/a, 90127 Palermo, Italy; alfonsolarosa@libero.it; 7 Department AGRARIA, Mediterranean University of Reggio Calabria, 89122 Reggio Calabria, Italy; vla.laface@unirc.it; 8 Independent Researcher, Via Torrente Allume, 6/A, 98027 Roccalumera (ME), Italy; fabio.luchino@tin.it; 9 Independent Researcher, Via Vittorio Emanuele III 41, 09020 Villanovaforru (SU), Italy; fr.maxia@gmail.com; 10 Independent Researcher, Via Pegorina 548, 35040 Casale di Scodosia (PD), Italy; luca.tosetto89@gmail.com

**Keywords:** mediterranean flora, biodiversity records, distribution range, exotic species, floristic records, invasive plants, regional flora, species occurrence, biological invasions, alien species management

## Abstract

In this paper, based on fieldwork and herbaria surveys, new data concerning the presence of 32 native and alien vascular species for Sicily (Italy) are provided. Among the native species, the occurrence of the following *taxa* is reported for the first time or confirmed after many decades of non-observation: *Aira multiculmis*, *Arum maculatum*, *Carex flacca* subsp. *flacca*, *Mentha longifolia*, *Oxybasis chenopodioides*, *Najas minor* and *Xiphion junceum*. Furthermore, we document the presence of three native species (*Cornus mas*, *Juncus foliosus* and *Limonium avei*) that, despite being repeatedly observed in Sicily and reported in the literature, are inexplicably omitted by the most recent authoritative checklists regarding the flora of Italy. Finally, fifteen alien species new to Sicily (including one new to Europe, i.e., *Pyrus betulifolia*) are reported and seven poorly documented allochthonous taxa are confirmed for the island, and for two of them, a status change is proposed. These new or confirmed records allow us to better define the European and national distribution of the targeted *taxa* and offer new insights on the native and alien flora of Sicily.

## 1. Introduction

During floristic and vegetational research carried out by the authors throughout the whole territory of Sicily, some taxa that were previously never reported or whose presence in Sicily was doubtful were recorded. After the latest checklists of the Sicilian [1,2] and Italian native and alien vascular flora [3,4,5], several new contributions have been published for Sicilian flora. Some of these refer to newly described endemic species [6,7,8,9,10,11,12,13,14,15,16,17,18,19,20,21,22], while others confirm or record for the first time species native to the island [23,24,25,26,27,28,29,30,31,32]. However, the large majority of these contributions consist of reports of exotic species [33,34,35,36,37,38,39,40,41,42,43,44,45,46,47,48,49,50,51,52,53,54,55,56,57,58]. In fact, as highlighted by Guarino et al. [59], Sicily, due to its long-lasting human history and central location in the Mediterranean basin, is still today a territory strongly subject to an intense and frequent introduction of exotic species for various purposes or accidentally. At the same time, although the native flora of Sicily is quite well known and its territory is one of the best-known floristic areas among Italian and Mediterranean regions [60], not all of the island has been investigated with the same level of effort, particularly in the case of the inland areas of central Sicily. Therefore, an extensive exploration of these less studied areas yielded the discovery or recent confirmation of some native *taxa* already recorded in other Italian regions or countries. Moreover, the examination of environments that are still less investigated, such as the numerous artificial basins and tanks found in Sicily, can lead to the discovery of new, mostly alien taxa. A systematic exploration of these environments was recently carried out through the MedIsWet project [61]. At the same time, the study of the taxonomic position of some critical taxa or populations allows us to identify and describe new taxa. Additional floristic data are collected through the activity of amateur botanists and plant enthusiasts on social networks, in some cases acting as veritable aggregators for botanical resources [62,63,64]. In particular, fundamental support for floristic research is currently provided by the members of the Facebook group “Flora spontanea Siciliana”, distributed throughout the regional territory.

The aim of this paper is to inform the scientific community about several unpublished new records, partly retrieved from social networks and validated by experts, related both to the native and alien flora of Sicily. In particular, 17 *taxa* are recorded for the first time in Sicily, while 13 other species, whose status was doubtful at the regional level, are here confirmed.

## 2. Results

**Figure 1:**
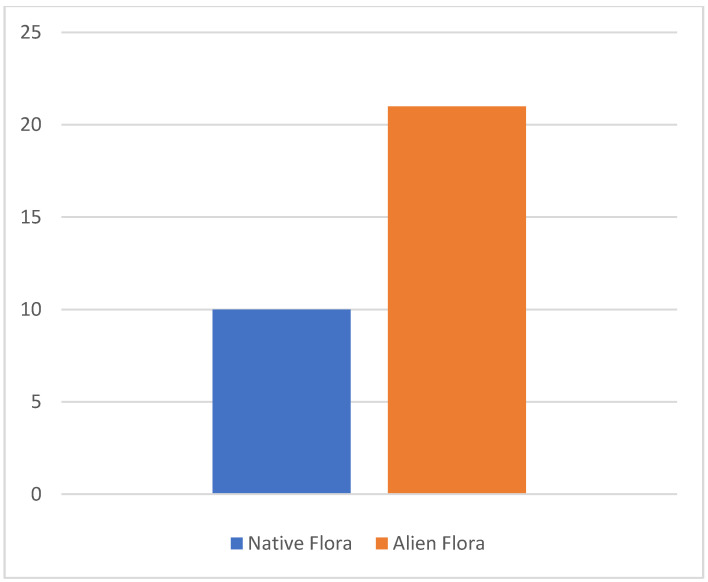
Percentage of native and alien species reported in the paper.

**Figure 2:**
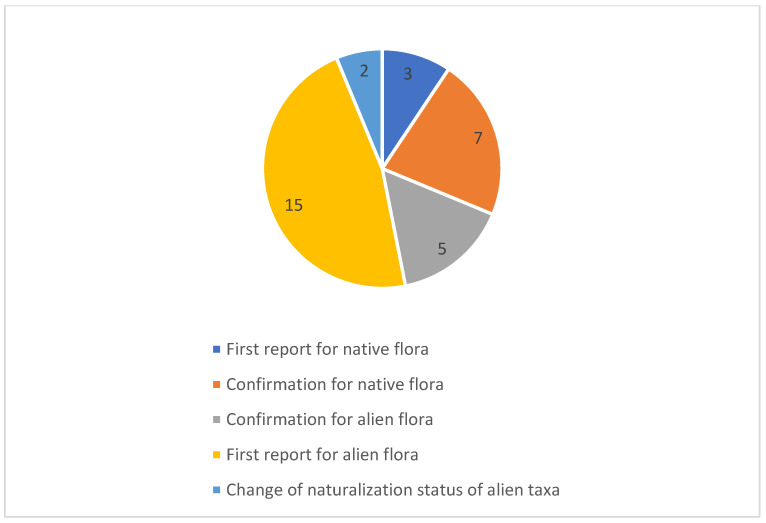
Typology of species recorded in this contribution.

**Figure 3:**
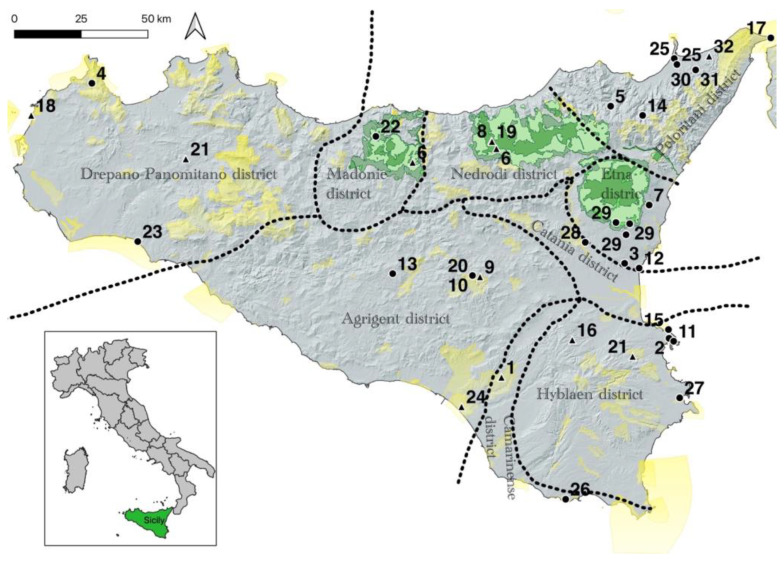
Distribution map of the new records. Black dot, alien species; black triangle, native species; yellow polygons, Natura 2000 areas; green polygons, park boundaries. 1. *Aira multiculmis*. 2. *Allium tuberosum*. 3. *Albuca canadensis*. 4. *Ambrosia artemisifolia*. 5. *Amorpha fruticosa*. 6. *Arum maculatum*. 7. *Bergenia crassifolia*. 8. *Carex flacca* subsp. *flacca*. 9. *Cornus mas*. 10. *Cydonia oblonga*. 11. *Fagopyrum esculentum*. 12. *Grevillea robusta*. 13. *Honorius nutans*. 14. *Hylotelephium spectabile*. 15. *Impatiens balsamina*. 16. *Juncus foliosus*. 17. *Kalanchoe laxiflora*. 18. *Limonium avei*. 19. *Mentha longifolia*. 20. *Morus alba*. 21. *Najas minor*. 22. *Nymphaea x marliacea*. 23. *Oenothera lindheimeri*. 24. *Oxybasis chenopodioides*. 25. *Passiflora morifolia*. 26. *Phacelia tanacetifolia*. 27. *Pontederia crassipes*. 28. *Pyrus betulifolia*. 29. *Secale cereale*. 30. *Solidago gigantea*. 31. *Symphyotrichum x salignum*. 32. *Xiphion junceum*.

The results bring to light new floristic data concerning 32 taxa for Sicily (Figure 1), of which 10 are native and 22 are non-native vascular species. Overall, from a chorological viewpoint, most species show an Asian and American distribution (31–25%, respectively), while the most represented life forms are therophytes (28%), phanerophytes (25%) and geophytes (22%). If we only consider alien species (22 sp.), the dominant life forms correspond to phanerophytes and therophytes (32–23%, respectively), while the most represented chorotypes are confirmed as Asian and American (45–36%, respectively) (Figure 2). Regarding the native species (10 sp.), the dominant life forms correspond to therophytes and geophytes (40–30%, respectively), while the most represented chorotype is Mediterranean and European (30%). Overall, the highest number of species recorded falls within the Peloritani (22%), Hyblaean (19%) and Agrigento (16%) districts (Figure 3). The phytogeographic districts most affected by alien plant species are the Peloritani (27%) and Hyblaean and Etna (18%) districts, while affected by native species are the Nebrodi (28%) and Agrigento and Hyblaean (18%) districts. The natural habitats most affected by alien plants are uncultivated areas (45%) and wetlands (28%), with the latter affected by the following phytosociological classes: *Nerio-Tamaricetea africanae*, *Phragmito-Magnocaricetea*, *Potametea pectinati*, *Saginetea maritimae*, *Isoeto-Nanojuncetea* and *Salici-Populetea nigrae*.

### Floristic Records

***Aira multiculmis*** Dumort.
Poaceae—T (scap)—Subtrop.—Native.
*Confirmation for Sicily*
*Exsiccatum*. Bosco di Santo Pietro (Caltagirone), contrada Ogliastro, sandy soils, 200–250 m a.s.l., 18.04.2022, leg. and det. D. Azzaro, *s.n.* (CAT!).

This is a species related to *Aira caryophyllea* L., which, in Italy, is known for its presence in Tuscany, Campania, Calabria and Sardinia [4]. Its occurrence in Sicily had been mentioned by Lojacono Pojero [65], while more recently, it was excluded by Bartolucci et al. [4] or considered doubtful by Giardina et al. [1]. The species was found in a fairly significant population on the sandy soils occurring in the clearings of the cork oak forest of Santo Pietro near Caltagirone, within the Camarino-Pachinense district, but on the basis of current knowledge, it is very localized. From a phytosociological point of view, the species grows in annual acidophilous meadows referable to the class *Helianthemetea guttate* Rivas Goday & Rivas-Mart. 1963. In the same area, there are two other species of *Aira*, from which *A. multiculmis* is distinguished by certain morphological characteristics. In particular, *A. caryophyllea* L. is characterized by spikelets, generally > 2.6 mm, and by the inferior glume diverging from the peduncle by less than 80° and by a smaller number of culms. *A. cupaniana* Guss. differs instead with its decidedly rough sheaths, smaller spikelets, sharply enlarged peduncles at the apex and its 2–2.5 mm glumes [66]. The population falls within the Nature Reserve “Riserva Naturale Orientata Bosco di Santo Pietro” and the SAC “Bosco di Santo Pietro” (ITA070005).

**Figure A1:**
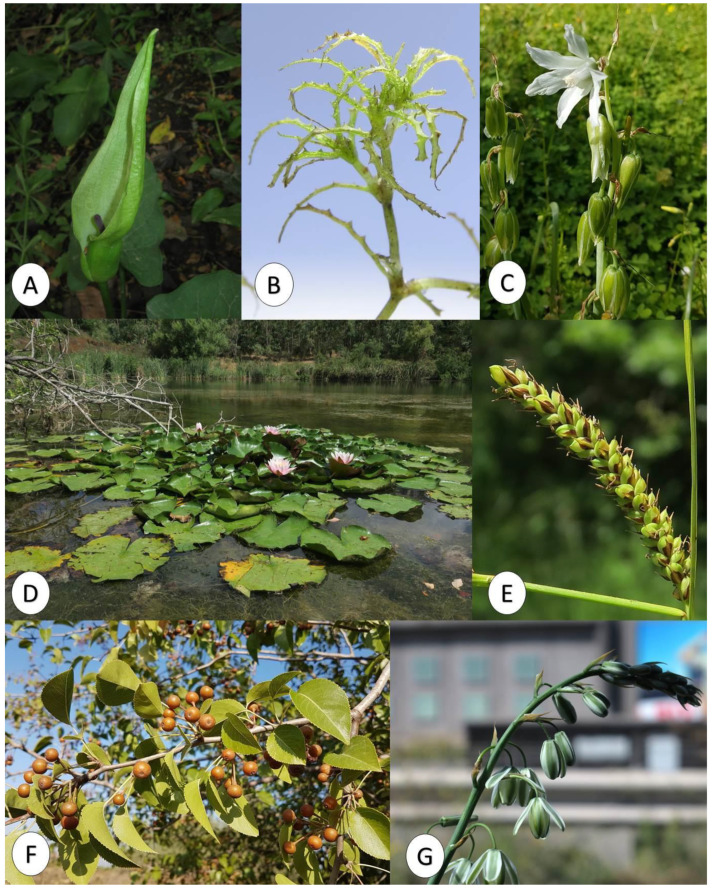
(**A**) *Arum maculatum* (photo by Alfonso La Rosa). (**B**) *Najas minor* (photo by Fabio Luchino). (**C**) *Honorius nutans* (photo by Antonia Cristaudo). (**D**) *Nymphaea x marliacea* (photo by Salvatore Cambria). (**E**) *Carex flacca* subsp. *flacca* (photo by Salvatore Cambria). (**F**) *Pyrus betulifolia* (photo by Antonia Cristaudo). (**G**) *Albuca canadensis* (photo by Salvatore Cambria).

***Albuca canadensis*** (L.) F.M. Leight (Figure A1G)
Asparagaceae—G (rhiz)—S-Afric.—Alien.
*Confirmation for Sicily and Italy*
*Exsiccatum*. Tangenziale di Catania, vicino all’uscita per Misterbianco, roadsides, 100 m a.s.l., 12.04.2022, leg. and det. S. Cambria, *s.n.* (CAT!).

This South African species was recorded for the first time in Italy by Nicolosi-Roncati [67] (sub *Albuca altissima* Dryand.), who reported its naturalization from a ruderal stand inside Catania. This author claims that the species was introduced from the local botanical garden and from there it spread to other areas of the city. Afterwards, the species was no longer recollected and was reported as dubious for Sicily and Italy by various authors [1,5]. We found a large population of *A. canadensis* growing on the roadside of the Catania ring road, consisting of almost 100 individuals. It is often found in dry grasslands with *Hyparrhenia hirta* (L.) Stapf., *Isatis tinctoria* L., *Ferula communis* L. and other typical species of the *Lygeo sparti-Stipetea tenacissimae* Rivas-Martínez 1978 class. Here, the presence of this taxon is confirmed for Italy and Sicily. We can state that the species does not represent a threat to the natural habitat 6220* “Pseudo-steppe with grasses and annuals of the *Thero-Brachypodietea*”. The population is circumscribed and does not show an increasing trend to expand into natural environments.

***Allium tuberosum*** Rottler ex Spreng.
Alliaceae—G (bulb)—Asia—Alien.
*New casual alien species for Sicily*
*Observatum*. Roadsides, Augusta (Syracuse), 10 m a.s.l., 09.10.2020, obs. R. Romano, det. F. Luchino.

A small population of this species was found in the suburb pavements of Augusta (SE Sicily), probably having escaped from nearby gardens. This species was already known in several regions in Northern and Central Italy [5].

***Ambrosia artemisifolia*** L.
Asteraceae—T (scap)—N. Amer.—Alien.
*New alien species for Sicily*
*Observatum*. Castelluzzo, San Vito Lo Capo (Trapani), 150 m a.s.l., 15.07.2020, obs. S. Montoleone, det. R. Guarino.

This North American species was observed in very anthropized environments near San Vito Lo Capo (western Sicily). Although it is still very rare in Sicily, its potential for further spread should be monitored, as it also represents one of the main causes of seasonal respiratory allergy in many European countries and in some areas of northern Italy [68]. In Italy, the species is known in most regions, excluding Sardinia, Umbria, Campania and Basilicata [5].

***Amorpha fruticosa*** L.
Fabaceae—P (caesp)—N. Amer.—Alien.
*New alien species for Sicily*
*Observatum*. Valle del Timeto, San Piero Patti-Librizzi, 450 m a.s.l., 23.05.2013, obs. and det. G. Torre.

This species was already known in all Italian regions as invasive or naturalized, except for Sicily [5]. Our finding extends its presence to Sicily, where a fairly significant population was surveyed in the north-eastern part of the island, between Librizzi and San Pietro Patti (Messina) along the edge of a stream among the riparian vegetation of *Salici purpureae-Populetea nigrae* Rivas-Martínez & Cantó ex Rivas-Martínez, Báscones, T.E. Díaz, Fernández-González & Loidi 2001. The plant community in which *A. fruticosa* grows is referable to habitat 92A0 “Salix alba and Populus alba galleries”.

***Arum maculatum*** L. (Figure A1A)
Araceae—G (rhiz)—Europ.—Native.
*New record for Sicily*
*Exsiccatum*. Piano Caterineci (Madonie, Palermo), orophilous vegetation with *Juniperus communis* subsp. *hemisphaerica*, 1590 m a.s.l., 31.05.2016, leg. V. Ilardi and S. Cambria, det. S. Brullo, *s.n.* (CAT!).
*Observatum*. Bosco della Tassita (Nebrodi, Caronia), Mesophilous woods, 1600 m a.s.l., 23.05.2013, obs. and det. A. La Rosa.

According to Bartolucci et al. [4], *Arum maculatum* occurs in almost all Italian regions, while it is absent in Sicily and doubtful for Sardinia. Giardina et al. [1] report the species as doubtful in Sicily, without providing any precise location. It is possible that in the past the species was confused with the more common *Arum cylindraceum* Gasparr., occurring in nearby localities. However, the two species are distinguished quite easily by the morphology of the underground organs, as *A. maculatum* has an ovoid tuberous rhizome, longer than broad and horizontally developing, while *A. cylindraceum* is characterized by a circular “bulb”, from the center of which the scape develops [69]. Pignatti [70] and Pignatti et al. [71,72,73,74] reported a peculiar form of *A. maculatum* for Sicily, named *nigro-maculatum* Fiori and indicated it for Caltanissetta. However, according to Sortino [75], it falls within the intraspecific variability of *Arum italicum*. Even the bioclimatic features of the aforementioned locality seem to be favorable to this arrangement, since *A. italicum* is widespread in Sicily at low elevations, while *A. maculatum* was observed by us only within the supra-Mediterranean bioclimatic belt of the Madonie and Nebrodi mountains in the mesophilous beech forests belonging to *Querco roboris-Fagetea sylvaticae* Br.-Bl. & Vlieger in Vlieger 1937 or orophilous shrublands dominated by *Juniperus communis* L. subsp. *hemisphaerica* (J. Presl & C. Presl) Nyman (*Pino sylvestris-Juniperetea sabinae* Rivas-Martínez 1965 class). The two populations fall within protected areas, such as the Madonie regional park and the Nebrodi regional park, as well as in the SAC Pizzo Fau, Monte Pomiere, Pizzo Bidi and Serra della Testa (ITA030014) and Parco delle Madonie (ITA020050).

**Figure A2:**
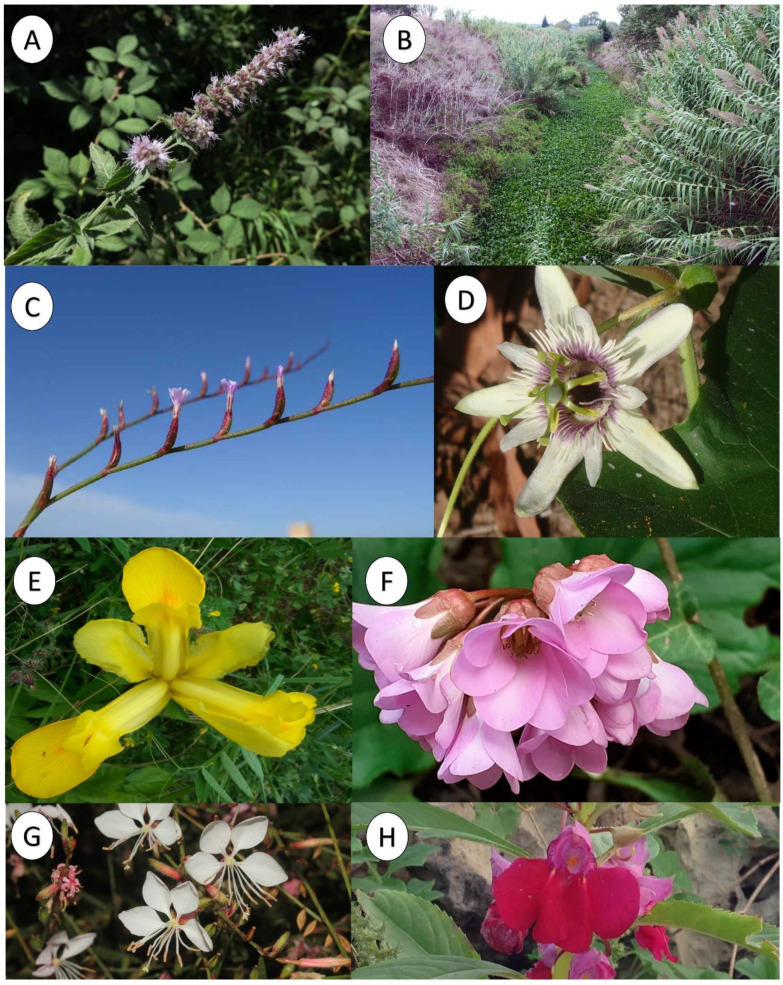
(**A**) *Mentha longifolia* (photo by Salvatore Cambria). (**B**) *Pontederia crassipes* (photo by Riccardo Guarino). (**C**) *Limonium avei* (photo by Michele Aleo). (**D**) *Passiflora morifolia* (photo by Antonia Cristaudo). (**E**) *Xiphion junceum* (photo by Antonino Scoglio). (**F**) *Bergenia crassifolia* (photo by Mauro Pappalardo). (**G**) *Oenothera lindheimeri* (photo by Orazio Caldarella). (**H**) *Impatiens balsamina* (photo by Caterina Arcidiacono).

***Bergenia crassifolia*** (L.) Fritsch. (Figure A2F)
Saxifragaceae—G (rhiz)—Asia—Alien.
*New alien species for Sicily*
*Observatum*. Monacella di Santa Venerina (Catania), 450 m a.s.l., 01.02.2022, obs. M. Pappalardo, det. O. Caldarella.

A few individuals of this ornamental species, quite frequent in the gardens of Sicily, were found as casual aliens near the small village of Monacella (Catania), on the eastern flank of Mt. Etna. Previously in Italy, it was reported only in some northern regions [5].

***Carex flacca*** Schreb. subsp. ***flacca*** (Figure A1E)
Cyperaceae—G (rhiz)—Europ.—Native.
*New record for Sicily*
*Exsiccatum*. Serra della Testa, hygrophilous vegetation with *Thelypteris palustris*, 1050 m a.s.l., 17.06.2021, leg. and det. S. Sciandrello, P. Minissale, D. Azzaro, S. Cambria, *s.n.* (CAT!).

*Carex flacca* subsp. *erythrostachys* (Hoppe) Holub is a common species in Sicily; however, as regards the subsp. *flacca*, there are only a few doubtful records mentioned by Lojacono Pojero [65] for various mountain localities and, more recently, by Raimondo [76] for Piano Battaglia (Madonie). However, the species is considered doubtful by Giardina et al. [1] and absent in Sicily by Bartolucci et al. [4]. Our field investigations allowed us to find a new stand in the regional park of Nebrodi mountains, where it grows in a peculiar hygrophilous vegetation dominated by *Thelypteris palustris* Schott. (*Thelypterido palustris-Caricetum paniculatae*, included in the *Caricion gracilis* alliance), recently surveyed by Sciandrello et al. [29].

***Cornus mas*** L.
Cornaceae—P (caesp/scap)—Eurimedit.—Native.
*Confirmation for Sicily*
*Exsiccatum*. Valley below Monte Canalotto, Piazza Armerina, riparian woods, 750 m a.s.l., 22.10.2021, leg. D. Azzaro, S. Cambria and det. S. Cambria, G. Tavilla, *s.n.* (CAT!).

The presence of *Cornus mas* in Sicily had already been reported by Giardina et al. [1] in certain localities in central Sicily and in some narrow valleys of the Erei mountains. Similarly, it is reported as “rare and probably only introduced (in reforestations)” in Sicily by Pignatti et al. [71,72,73,74]. Despite this, Bartolucci et al. [4] do not report it for Sicily. Our finding testifies to its presence in the previously mentioned area, where it often grows in association with *Cornus sanguinea* L., though much less frequently. From a phytosociological point of view, it grows in the riparian forest of *Salici purpureae-Populetea nigrae* Rivas-Martínez & Cantó ex Rivas-Martínez, Báscones, T.E. Díaz, Fernández-González & Loidi 2001. The surveyed population falls within the SAC “Vallone Rossomanno” (ITA060010) and in the regional Nature Reserve “Riserva Rossomanno-Grottascura-Bellia”.

***Cydonia oblonga*** Mill.
Rosaceae—P (scap)—W Asia—Alien.
*Confirmation for Sicily as a casual alien*
*Exsiccatum*. Valley below Monte Canalotto, Piazza Armerina, riparian woods, 750 m a.s.l., 22.10.2021, leg. D. Azzaro, S. Cambria and det. S. Cambria, G. Tavilla, *s.n.* (CAT!).

The naturalization of this species in Sicily had already been reported by Terpò [77], Giardina et al. [1] and Pignatti et al. [72]. However, it is not reported in the recent checklists regarding the Italian flora [4,5]. Our findings confirm the presence of this species as a casual alien in Sicily. In fact, a few young trees, most likely seed-generated, have been found within a riparian wood of *Salici purpureae-Populetea nigrae* class (referable to habitat 92A0 “Salix alba and Populus alba galleries”), protected in the regional Nature Reserve “Riserva Rossomanno-Grottascura-Bellia” and in the SAC “Vallone Rossomanno” (ITA060010). However, the few *C. oblonga* individuals do not currently represent a real threat to this habitat.

***Fagopyrum esculentum*** Moench
Polygonaceae—T (scap)—Central Asia—Alien.
*Confirmation for Sicily*
*Observatum*. Augusta, ex Saline Regina (Siracusa), Roadsides, 10 m a.s.l., 15.10.2021, obs. and det. R. Romano, A. La Rosa.

*Fagopyrum esculentum* is not reported for Sicily in any recent work concerning the vascular flora of Sicily [1,4,5,71,72,73,74] and historical records regarding its presence are lacking. However, it was recorded in a list of alien species in Sicily by Raimondo et al. [78], without specifying any precise location, as a cultivated and rarely naturalized species. Our findings near Augusta (E Sicily) confirm this species as a casual alien in Sicily. The site falls within the SAC “Saline di Augusta” (ITA090014).

***Grevillea robusta*** A. Cunn. ex R. Br.
Proteaceae—P (scap)—Australia—Alien.
*New casual alien species for Sicily*
*Observatum*. Catania, 10 m a.s.l., 24.10.2020, obs. and det. F. Gambilonghi.

This Australian species, commonly used for road tree planting over the last three decades, was found on the sidewalks and retaining walls of Catania. In Italy, it was already recorded as a casual alien in Lazio and Campania [5].

***Honorius nutans*** (L.) Gray (Figure A1C)
Asparagaceae—G (bulb)—W Asia—Alien.
*Confirmation for Sicily*
*Observatum*. C.da Serra Pantano, Caltanissetta, olive groves, 470 m a.s.l., 02.04.2018, obs. and det. A. Cristaudo.

The naturalization of this taxon in Sicily was observed only once by Lojacono Pojero [65] near Enna (Central Sicily). The new location was situated on the outskirts of Caltanissetta, where a small population of the species colonized part of an olive grove. The agricultural practices carried out in recent years have probably led to the disappearance of this population, but its presence in other places in the nearby areas cannot be excluded.

***Hylotelephium spectabile*** (Boreau) H. Ohba
Crassulaceae—H (scap)—Eurosiber.—Alien.
*New casual alien species for Sicily*
*Observatum*. Oak wood near San Basilio, Novara di Sicilia (Messina) 670 m a.s.l., 08.04.2018, obs. A. Ferrara Currò, det. O. Caldarella.

This species, commonly cultivated as an ornamental plant, was found in a deciduous oak wood (*Querco-Fagetea* class) in the territory of Novara di Sicilia (NW Sicily). In Italy it was already observed as a casual alien in Lombardy, Marche and Umbria [5]. According to the Directive 43/92/EEC, this species has been found in the habitat 91AA* “Eastern white oak woods”.

***Impatiens balsamina*** L. (Figure A2H)
Balsaminaceae—T (scap)—Asia—Alien.
*New record for Sicily*
*Observatum*. C.da Campolato, Augusta (Syracuse), dry grasslands, 10 m a.s.l., 02.10.2018, obs. C. Arcidiacono, det. O. Caldarella.

This species, often cultivated in gardens as a summer annual, was observed in a coastal grassland dominated by *Hyparrhenia hirta* (L.) Stapf (*Lygeo-Stipetea* class) on calcarenitic substrates near Augusta (SE Sicily). It was reported as a casual alien by Galasso et al. [5] for most of the northern regions as well as Lazio and Puglia. The natural habitat affected by this species is the 6220* “Pseudo-steppe with grasses and annuals of the Thero-Brachypodietea”.

***Juncus foliosus*** Desf.
Juncaceae—T (scap)—Stenomedit.—Native.
*Confirmation for Sicily*
*Exsiccatum*. Oxena river, near Militello Val di Catania (eastern Sicily), annual hygrophilous vegetation, 310 m a.s.l., 04.07.2021, leg. and det. D. Azzaro, *s.n.* (CAT!).

Bartolucci et al. [4] and Giardina et al. [1] report this species as doubtful for Sicily, while the records by Zodda [79], Lojacono Pojero [65], Barbagallo and Furnari [80] and Raimondo et al. [78] are considered questionable. Recently, Lastrucci et al. [81] considered it to no longer be present in Sicily. However, this species was recorded in several localities in Sicily by Pignatti et al. [71,72,73,74] and in the phytosociological relevés by Sciandrello et al. [82] from south-eastern Sicily. Our findings from Oxena river, near Catania, confirm the occurrence of this species in Sicily, where it is linked to the thero-hygrophilous communities belonging to *Isoëto-Nanojuncetea* Br.-Bl. & Tüxen ex Westhoff, Dijk & Passchier 1946 and *Saginetea maritimae* Westhoff, Leeuwen & Adriani 1962.

***Kalanchoe laxiflora*** Baker
Crassulaceae—T (scap)—S.-Afric., Madagascar—Alien.
*Confirmation as casual alien for Sicily*
*Observatum*. Via Fortino, Torre Faro, Messina, 10 m a.s.l., 23.10.2022, obs. and det. V.L.A. Laface.

The occurrence of this species as a casual neophyte in Europe and Italy has recently been confirmed by Stinca et al. [83] and Spampinato et al. [84], respectively, for Basilicata and Calabria. Previously, Galasso et al. [5] reported *K. laxiflora* doubtfully for Toscana and Sicily. Our findings confirm the presence of the species in this region, where it is occasionally cultivated. In fact, the observed population, consisting of a few individuals that probably escaped from nearby gardens, colonize wall cracks near the road, within the SPA “Monti Peloritani, Dorsale Curcuraci, Antennamare e area marina dello Stretto di Messina” (ITA030042).

***Limonium avei*** (De Not.) Brullo & Erben (Figure A2C)
Plumbaginaceae—T (scap)—W-Medit.—Native.
*Confirmation for Sicily*
*Exsiccatum*. Saline di Nubia presso Paceco (Trapani), 10 m a.s.l., 30.04.2022, leg. and det. M. Aleo (CAT!).
*Observatum*. Isola Grande dello Stagnone (Marsala), wet clay soils, 5 m a.s.l., 02.03.2022, obs. and det. S. Cambria and G. Tavilla.

The presence of this species has not been recorded in Sicily by Bartolucci et al. [4], despite many authors [1,85,86,87,88,89,90] repeatedly recording several stands in western Sicily and Lampedusa. Here, we confirm the occurrence of the species in the coastal brackish environments between Trapani and Marsala (western Sicily) and also in the Isola Grande dello Stagnone, inside the regional Nature Reserve “Riserva Saline di Trapani e Paceco” and the SPA “Stagnone di Marsala e Saline di Trapani” (ITA010028), where it is widespread. This annual halophyte prefers soils that has a high salt concentration and is rich in nitrates and grows in markedly xeric environmental conditions, together with several species of the *Limonion avei* alliance (*Saginetea maritimae*).

***Mentha longifolia*** (L.) L. (Figure A2A)
Lamiaceae—H (scap)—Paleotemp.—Native.
*New record for Sicily*
*Exsiccatum*. Serra della Testa (Nebrodi), hygrophilous vegetation, 1000 m a.s.l., 17.06.2021, leg. and det. S. Sciandrello, P. Minissale, D. Azzaro, S. Cambria, s.n. (CAT!).

The species is not mentioned by Giardina et al. [1], while it is recorded from Sicily by Conti et al. [3]. Bartolucci et al. [4] reported the reference for Sicily as an incorrect record. Our field surveys have resulted in the discovery of the species in the Nebrodi area, where the species is quite widely distributed and overlooked, perhaps due to the confusion with *M. spicata* L., from which it is mainly distinguished by the presence of only simple straight hairs and lanceolate leaves, with maximum width towards the median part [71,72,73,74]. The species is linked to the *Phragmito australis*-*Magnocaricetea elatae* Klika in Klika & Novák 1941 communities and the surveyed population falls within the Nebrodi Regional Park.

***Morus alba*** L.
Moraceae—P (scap)—E Asia—Alien.
*New record for Sicily as casual alien*
*Exsiccatum*. Valley below Monte Canalotto, Piazza Armerina, riparian woods, 750 m a.s.l., 22.10.2021, leg. D. Azzaro, S. Cambria and det. S. Cambria, G. Tavilla, *s.n.* (CAT!).

Despite being commonly cultivated, this species has never been ascribed to the allochthonous flora of Sicily. Based on the observation of some young individuals in the riparian vegetation dominated by *Populus nigra* and *P. alba* (*Salici purpureae-Populetea nigrae* class, habitat 92A0 “Salix alba and Populus alba galleries”) colonizing a narrow valley in central Sicily, we report for the first time *Morus alba* as a casual alien for the Sicilian flora. This location is protected in the regional Nature Reserve “Riserva Rossomanno-Grottascura-Bellia” and in the SAC “Vallone Rossomanno” (ITA060010).

***Najas minor*** All. (Figure A1B)
Hydrocharitaceae—I (rad)—Paleotemp.—Native.
*New record for Sicily*
*Exsiccatum*. Artificial basin of Cozzo Ogliastri, 381 m a.s.l., 30.07.2021, leg. and det. L. Tosetto, F. Luchino, S. Cambria, s.n. (CAT!).
*Observatum*. Artificial basin near Roccamena, 470 m a.s.l., 02.04.2018, obs. and det. S. Costa, O. Caldarella and A. La Rosa.

Conspicuous populations of this species were found in two artificial ponds near Roccamena (Palermo) and in a small water reservoir near Melilli (Syracuse). It constitutes a dense, monophytic aquatic vegetation with a summer optimum. Considering that the species has never been reported for Sicily and its presence seems so far restricted to man-made habitats, it is possible to hypothesize its relatively recent arrival on the island. According to Volk [91], the spread of this species is related to water birds and, in particular, to ducks, as the seeds of *Najas minor* partly retain their vitality after digestion and can germinate in polluted waters. In Italy, *Najas minor* is recorded only in northern and central regions, as well as in Sardinia [4].

***Nymphaea x marliacea*** Lat.-Marl. (Figure A1D)
Nymphaeaceae—I (rad)—horticultural origin—Alien.
*New record for Sicily as casual alien*
*Exsiccatum*. Artificial pond in Contrada Cella di Fico near Isnello (Palermo), 742 m a.s.l., 14.08.2019, leg. and det. S. Cambria, s.n. (CAT!).

This horticultural hybrid was observed in an artificial pond, built as a water reservoir for the irrigation of nearby agricultural lands in the 1970s and today is in disuse, located within private property occupied by a livestock farm. Over the years, the basin became naturalized and has been colonized by various aquatic and hygrophilous species, such as *Potamogeton pusillus, Scirpoides holoschoenus, Juncus inflexus, Juncus effusus* and *Epilobium hirsutum*, characterizing an aquatic community of *Potametea pectinati* Klika in the Klika & Novák 1941 class. The origin of this *Nymphaea* population is somewhat uncertain, since the site has been abandoned for many years and the distance from the inhabited centers and gardens is quite significant. In any case, the taxon at issue belongs to a complex of horticultural hybrids, whose putative parental species are *N. alba* L., *N. mexicana* Zucc. and *N. odorata* Aiton [92]. Based on multiple years of observation, this population is spontaneously expanding within the basin. In Italy, *N. x marliacea* has been so far reported as a casual alien only in northern regions, such as Lombardia and Trentino-Alto Adige [5,93]. It must be highlighted that the discovered population is located within the Madonie regional park and the SPA “Parco delle Madonie” (Parco delle Madonie).

***Oenothera lindheimeri*** (Engelm. & A. Gray) W.L. Wagner & Hoch (Figure A2G)
Balsaminaceae—H (bienn)—N. Amer.—Alien.
*Change of status from casual alien to naturalized*
*Observatum*. Lido Fiori beach near Menfi (Agrigento), 10.08.2022, obs. and det. O. Caldarella.

*Oenothera lindheimeri* was reported for the first time in Sicily as a casual species by Galasso et al. [39]. Following the observations carried out in the last 5 years on the consistent population occurring near the beach of Lido Fiori near Menfi (Agrigento), the change of status from casual to naturalized is here proposed. Therefore, the species represents a serious threat to the local psammophilous vegetation, referable to the *Euphorbio paraliae-Ammophiletea australis* Géhu & Rivas-Martínez in Rivas-Martínez, Asensi, Díaz-Garretas, Molero, Valle, Cano, Costa & Díaz 2011 (habitat 2120 “Shifting dunes along the shoreline with *Ammophila arenaria* (white dunes)”).

***Oxybasis chenopodioides*** (L.) S. Fuentes, Uotila & Borsch.
Chenopodiaceae– T (scap)—Subcosmop.—Native.
Confirmation for Sicily.
*Exsiccatum*. Biviere di Gela (southern Sicily), lakeshores, 10 m a.s.l., 07.08.2002, leg. and det. S. Sciandrello and S. Brullo, s.n. (CAT!).

This species was reported for the first time in Sicily (sub *Chenopodium botryoides* Sm.) by Brullo and Sciandrello [94] for the lake “Biviere di Gela” (southern Sicily), within the nature reserve Biviere di Gela and the SPA “Torre Manfria, Biviere e Piana di Gela” (ITA050012). However, this record was ignored by Giardina et al. [1], Raimondo et al. [2,95] and Bartolucci et al. [4]. Iamonico [96], while not citing this report for the Biviere di Gela, attributed an herbarium specimen collected in the 19th century near Messina to *Oxybasis chenopodioides* but hypothesized its regional extinction due to the age of the collected specimen and the current dense urbanization of this locality. Therefore, based on our data, it is possible to confirm the current presence of this species in Sicily, where the species is linked to thero-hygrophilous vegetation with the summer–autumn development of *Nanocyperetalia flavescentis* Klika 1935.

***Passiflora morifolia*** Mast. (Figure A2D)
Passifloraceae—P (lian)—S. Amer.– Alien.
*New naturalized alien species for Sicily*
*Exsiccatum*. Milazzo, presso l’Istituto Professionale Agrario (IPSAA), 10 m a.s.l., 03.07.2010, leg. and det. A. Cristaudo, *s.n.* (CAT!).
*Observatum*. Piana di Milazzo, nelle vicinanze dei Vivai Torre, 40 m a.s.l., 22.06.2022, obs. and det. G. Torre.

This species, native to tropical America, is cultivated in gardens for its ornamental qualities. Its ability to disseminate itself is well known among gardening enthusiasts and, in fact, the presence of the species as a casual alien has been already reported in Sardinia by Galasso et al. [36]. According to our observations, it is quite frequent in the irrigated land of the Milazzo plain (Messina), particularly near gardens and nurseries, and its presence has now been well established in the territory for more than 10 years.

***Phacelia tanacetifolia*** Benth.
Hydrophyllaceae—T (scap)—N. Amer.– Alien.
*New casual alien species for Sicily*
*Observatum*. Marina di Modica (Ragusa), 5 m a.s.l., 16.04.2020, obs. C. Spadaro, det. R. Guarino.

This North American species, adventitious in almost all regions of central-northern Italy, including Sardinia [5], is sometimes cultivated as an ornamental nectar plant and tends to escape cultivation in disturbed environments. It was found in uncultivated land near Marina di Modica (SE Sicily), where the species is, however, represented only by a small population.

***Pontederia crassipes*** Mart. (Figure A2B)
Pontederiaceae—I (rad)—S. Amer.—Alien.
*Change of status from casual alien to invasive*
*Exsiccatum.* Canalizzazioni del Fiume Ciane (Siracusa), 5 m a.s.l., 21.09.2006, leg. and det. R. Guarino, s.n. (PAL!).

This species was recorded for the first time in Sicily by Bartolo et al. [97], who observed it in a channel connecting the Pantano Gariffi and the sea in SE Sicily (Ispica, Ragusa province). Since then, *P. crassipes* has been repeatedly reported as a casual alien in Sicily [1,5,71,72,73,74]. Following the observations carried out in the last 16 years on the abundant population extant in the channels of the Ciane River (SE Sicily, Syracuse Province), the change of status from casual to invasive is here proposed. The surveyed site falls within the nature reserve “Fiume Ciane e Saline di Siracusa” and in the SAC “Saline di Siracusa e Fiume Ciane” (ITA090006). This species represents a significant threat to the habitat 3260 “Water courses of plain to montane levels with the *Ranunculion fluitantis* and *Callitricho-Batrachion* vegetation”.

***Pyrus betulifolia*** Bunge (Figure A1F)
Rosaceae—P (caesp/scap)—E Asia—Alien.
*New record for Sicily and Europe as a casual alien*
*Exsiccatum*. Paternò (Catania), near Pietralunga bridge, scrublands, 89 m a.s.l., 15.10.2019, leg. and det. A. Cristaudo, F. Carruggio, *s.n.* (CAT!).

*Pyrus betulifolia* is a wild pear native to eastern Asia and, in particular, to north-central and south-east China, Laos, Manchuria and Tibet [98]. According to literature data, the occurrence of this species in the exotic flora of Europe and Italy had never been reported. Outside its original range, the species has been naturalized only in Illinois, United States of America [99]. Moreover, *P. betulifolia* is listed in the Invasive Plant Atlas of the US, but it does not appear to be a species with significant invasive potential [100]. According to Swearingen et al. [101], seeds can be dispersed from planted trees via birds. The surveyed population was small, consisting of only three shrubs that grow within a long-abandoned agricultural area, subject to flooding during the winter and autumn periods. In general, *P. betulifolia* occurs within very scattered thermo-hygrophilous vegetation with *Tamarix africana* Poir. and *T. gallica* L. (*Nerio-Tamaricetea africanae* Br.-Bl. & O. Bolòs 1958 class, habitat 92D0 “Southern riparian galleries and thickets (*Nerio-Tamaricetea* and *Securinegion tinctoriae*)”), which occurs also in the nearby areas next to the Simeto river. The origin of this population can almost certainly be related to the use of this species as rootstock for ‘Coscia’ pear (*Pyrus communis*), as reported by Stern et al. [102] for Israel, although the species is rarely offered in the catalogs of Italian nurseries. Currently, this type of cultivation has almost disappeared from this area, where citrus groves are predominant, while it is possible that in the past it was more widely practiced. The surveyed plants show different ages and bear fruit regularly. Further investigations are needed to understand the future dynamics of this small population.

***Secale cereale*** L. subsp. ***cereale***
Poaceae—H bienn—Asiat.—Alien.
*New record for Sicily as a casual alien*
*Observatum*. Loc. Contrada San Leo, Belpasso, 1000 m a.s.l.; loc. Caldera, Contrada Simita e Tarderia, Pedara, 830–870 m a.s.l.; Contrada Mompilieri, Nicolosi, 640 m a.s.l. (Catania), 27.07.2021, obs. F. Mascia and N. Serafica, det. F. Mascia.

In Sicily, the traditional cultivation of *Secale cereale* has been reported since the end of the XVIII century [103], mainly in the Aeolian islands and the mountain (Etna and Nebrodi) areas of the Catania and Enna provinces. This crop, in particular, a local variety called *Irmana*, had a substantial increase during the Second World War and was later almost completely abandoned [104] and was recently considered probably extinct [1]. In the last decade, the impulse to recover native cereal crops has led to the rediscovery of rye and its renewed cultivation, especially in the province of Catania [105]. In Italy, it is reported as a casual alien for all regions except Molise, Sicily and Val d’Aosta [3,106]. In Sicily, it was found along field edges and roadsides and secondarily as an occasional weed in *Lupinus albus* L. and *Vicia faba* L. fields.

***Solidago gigantea*** Aiton
Asteraceae—H (scap)—N. Amer.—Alien.
*New record for Sicily as a casual alien*
*Observatum*. Pace del Mela (Messina), 100 m a.s.l., 2.10.2022, obs. and det. F. Berenato.

This North American species is reported as casual or naturalized in the regions of central-northern Italy, Abruzzo and Calabria [5]. In Sicily, it was observed in seasonal humid environments with vegetation referable to *Scrophulario-Helichrysetea italici* Brullo, Scelsi & Spampinato 1998 near Pace del Mela (Messina), colonizing the edges of ditches and canals. This species was observed within the habitat 3250 “Constantly flowing Mediterranean rivers with *Glaucium flavum*”.

***Symphyotrichum x salignum*** (Willd.) G.L. Nesom
Asteraceae—NP—N. Amer.—Alien.
*New record for Sicily as a casual alien*
*Observatum*. Pace del Mela (Messina), 100 m a.s.l., 2.10.2022, obs. and det. F. Berenato.

*Symphyotrichum x salignum*, probably a natural hybrid between *S. novi-belgii* and *S. lanceolatum*, was surveyed in disturbed aspects of *Scrophulario-Helichrysetea* vegetation (habitat 3250 “Constantly flowing Mediterranean rivers with *Glaucium flavum*”) near Pace del Mela (Messina). In Italy, it was reported in most of the northern and central regions and Sardinia [5].

***Xiphion junceum*** (Poir.) Parl. (Figure A2E)
Iridaceae—G (bulb)—W-Stenomedit.—Native.
*Confirmation for Sicily*
*Observatum*. Olive Groves, Spadafora (Messina), 200 m a.s.l., 14.04.2015, obs. A. Scoglio, det. A. La Rosa.

This species, native to Iberian Peninsula, Northern Africa and Sicily [98], was recorded for the first time in Sicily by Gussone [85] on the southern coast between Palma di Montechiaro and Licata and later confirmed in the same locality by Ponzo [107]. The species was also reported in Riesi [79,108] and Mascali [78]. We confirm the presence of the species on the island and report it for the first time in northern Sicily. In particular, the plant was found near Messina in an olive grove, where the species is represented by a small population.

## 3. Materials and Methods

### 3.1. Study Area

With a surface of 25.711 km², Sicily represents the largest island in the Mediterranean Sea, located immediately south of the Italian Peninsula [109]. Sicily’s mountain ranges are mainly distributed along the northern sector of the island, namely the Madonie (reaching 1979 m a.s.l.), the Nebrodi (1847 m a.s.l.) and the Peloritani (1374 m a.s.l.). In the central and southern sectors, the landscape is mainly characterized by a typical low relief. The highest peak is the Etna volcano (3340 m). This considerable altitudinal heterogeneity encompasses several climate zones, from semi-arid to humid. Annual rainfall varies from 250 to 1400 mm, whereas the average temperature is 18 °C, with values below zero in the inland territory in winter and over 40 °C along the coast in summer. The smaller islands around Sicily are the Aeolian and the Aegadian archipelagos, as well as the Pelagie, Ustica and Pantelleria. According to the classification of Bazan et al. [109], Sicily is divided into the following 11 bioclimatic belts: 1. upper infra-Mediterranean (It 450–515, 0–30 m a.s.l.); 2. lower thermo-Mediterranean (It 400–450, 0–220 m a.s.l.; 3. upper thermo-Mediterranean (It 350–400, 0–450 m a.s.l.); 4. lower meso-Mediterranean (It 285–350, 250–700 m a.s.l.); 5. upper meso-Mediterranean (It 220–285, 620–1030 m a.s.l.); 6. lower supra-Mediterranean (It 150–220, 960–1400 m a.s.l.); 7. upper supra-Mediterranean (It 120–150, 1370–1550 m a.s.l.); 8. lower oro-Mediterranean (Tp 675–900, 1550–2000 m a.s.l.); 9. upper oro-Mediterranean (Tp 450–675, 2000–2400 m a.s.l.); 10. lower cryoro-Mediterranean (Tp 150–450, 2400–3000 m a.s.l.); and 11. upper cryoro-Mediterranean (Tp 1–150, above 3000 m a.s.l.).

According to the phytogeographic classification proposed by Brullo et al. [110] and subsequently modified [111], 14 well-defined districts can be identified within Sicily, distinguished by floristic and vegetational peculiarities, as well by remarkable geological, geomorphological and climatic features [109,112,113]. The research was carried out throughout all the districts of the island (Figure 3).

### 3.2. Data Sources

The floristic data are based on field investigations carried out from 2015 to 2022, herbaria surveys (CAT, PAL, FI, RO) and bibliographic analysis. The collected or examined plant materials are preserved mainly in CAT herbarium or private herbaria. Nomenclatures, taxonomic concepts and notes on the regional distribution are based mainly on the checklists published by Giardina et al. [1], Bartolucci et al. [4] and Galasso et al. [5], as well as on the online resources “FlorItaly—Portal to the Flora of Italy” (http://dryades.units.it/floritaly/index.php, accessed on 27 August 2022) and “Acta Plantarum” (https://www.actaplantarum.org, accessed on 27 August 2022). Syntaxonomic classification follows Mucina et al. [114].

Specimens were identified according to Pignatti et al. [71,72,73,74] or other monographic works cited in the notes for each species. In the results, *taxa* are arranged in alphabetical order. For each taxon, according to Pignatti et al. [71,72,73,74], the following data are reported: family, life form, native range, life form, chorology or origin, as well as the herbarium specimens and/or observations, phytosociological reference, phytogeographic district and occurrence in protected areas. The degree of naturalization was evaluated according to Galasso et al. [5]. Details on locality, habitat, altitude, date of collection or observation, names of collector/observer and identifier are also provided. Additionally, other data relating to the floristic record, any previous reports and other information on taxonomy, ecology and distribution are discussed in the notes.

## 4. Discussion and Conclusions

This paper presents new data concerning 32 taxa of native and non-native vascular species for Sicily (Figure 3). In particular, *Aira multiculmis*, *Arum maculatum* and *Najas minor* are recorded for the first time in the native flora of Sicily, while *Carex flacca* subsp. *flacca*, *Cornus mas*, *Juncus foliosus*, *Limonium avei*, *Mentha longifolia*, *Oxyasis chenopodioides* and *Xiphion junceum* are confirmed for the island, since their presence on the island, as indicated by some authors, was considered doubtful in the recent literature, mainly due to very old or vague reports. In regard to non-native species, the occurrence of *Albuca canadensis*, *Honorius nutans, Fagopyrum esculentum*, *Kalanchoe laxiflora* and *Cydonia oblonga* are confirmed after some decades. Finally, *Pontederia crassipes* is recorded as invasive and *Morus alba*, *Nymphaea x merlata*, *Allium tuberosum*, *Grevillea robusta*, *Ambrosia artemisifolia*, *Impatiens balsamina*, *Hylotelephium spectabile*, *Bergenia crassifolia*, *Secale cereale*, *Symphyotrichum x salignum*, *Solidago gigantea*, *Phacelia tanacetifolia*, *Passiflora morifolia*, *Pyrus betulifolia* and *Amorpha fruticosa* are recorded as casual aliens for the first time in the region. The new findings for the native flora confirm the remarkable floristic richness of the island, which, in spite of what can be hypothesized on the basis of the numerous floristic studies recently published on the Sicilian territory, is still worthy of further study. Furthermore, the results obtained in this research confirm the rapid increase in exotic species even in natural environments; thus, in some cases, the careful monitoring of species with greater invasive potential is advised. Particular attention must be paid to protected areas, such as the sites of the Natura 2000 network, mentioned several times here in the new records, where alien species can cause damage or alteration to the habitats of conservation importance [115].

Thus, having a continuous account of new records allows the planning of early preventive actions for their further diffusion, saving on future high management costs [116]. In conclusion, although attention is often focused on invasive alien species (IAS), causal alien species should not be neglected in the management of a territory, as they are quite difficult to control after their spread and change of status [117,118].

## Data Availability

Not applicable.
